# Prognostic value of baseline LIPI, LDH and dNLR in ES-SCLC patients receiving immune checkpoint inhibitors: a systematic review and meta-analysis

**DOI:** 10.3389/fimmu.2025.1640066

**Published:** 2025-09-30

**Authors:** Chenyi Zhou, Quanman Hu, Xiaoru Song, Xiyin Wang, Ran Kong, Fei Zhao, Boying Wu, Shuaiyin Chen, Bin Jia

**Affiliations:** ^1^ Department of Oncology, The First Affiliated Hospital of Zhengzhou University, Zhengzhou, Henan, China; ^2^ College of Public Health, Zhengzhou University, Zhengzhou, China

**Keywords:** lung immune prognostic index, lactate dehydrogenase, derived neutrophil-to-lymphocyte ratio, extensive-stage small cell lung cancer, immune checkpoint inhibitors, prognostic

## Abstract

**Background:**

Existing research presents conflicting findings on how baseline lung immune prognostic index (LIPI), lactate dehydrogenase (LDH), and derived neutrophil-to-lymphocyte ratio (dNLR) levels influence the prognosis of patients with extensive-stage small cell lung cancer (ES-SCLC) undergoing treatment with immune checkpoint inhibitors (ICIs). This meta-analysis aims to clarify their impact.

**Methods:**

A comprehensive search of published literature up to January 1, 2025 was conducted in PubMed, Web of Science, Cochrane Library, and Embase. The study evaluated the association between baseline LIPI, LDH, and dNLR levels and overall survival (OS) and progression-free survival (PFS) in ES-SCLC patients receiving ICIs. Subgroup analyses were performed based on relevant factors, and the study adhered to PRISMA 2020 guidelines.

**Results:**

This meta-analysis included 23 studies (LIPI: 10 studies/1,291 patients; LDH: 17 studies/1,768 patients; dNLR: 5 studies/324 patients). Elevated LIPI was significantly associated with poorer PFS (hazard ratio (HR) = 1.57, 95% confidence interval (95% CI) 1.20 - 2.06; I² = 59.0%, P = 0.013) and OS (HR = 1.76, 95% CI 1.26 - 2.45; I² = 64.2%, P < 0.001). Baseline LDH correlated with poorer OS (HR = 1.70, 95% CI 1.29 - 2.24; I² = 78.7%, P < 0.001), while elevated dNLR affected OS (HR = 2.05, 95% CI 1.02 - 4.12; I² = 86.31%, P < 0.001). Subgroup analysis showed that LIPI-PFS heterogeneity came from univariate and multivariate groupings. And LDH-OS heterogeneity was driven by country grouping.

**Conclusion:**

In ES-SCLC patients treated with ICIs, elevated baseline LIPI indicates reduced PFS and OS, while higher LDH and dNLR levels correlate with poorer OS. Monitoring these biomarkers can inform clinical decisions and enhance patient counseling.

**Systematic review registration:**

https://www.crd.york.ac.uk/PROSPERO/view/CRD420251123579, identifier CRD420251123579.

## 1 Introduction

Lung cancer is one of the most prevalent malignant tumors globally, characterized by high incidence and mortality rates (1, 2). Small cell lung cancer (SCLC) constitutes approximately 15% of all lung malignancies and is distinguished by rapid growth, early metastasis, aggressive invasiveness, and an unfavorable prognosis (3). Approximately two-thirds of small cell lung cancer patients are diagnosed after metastasis due to non-specific symptoms. This stage is called extensive stage and has a very poor prognosis (4), with a 5-year survival rate reported to be as low as 1-2% (5). Over the past three decades, platinum-based doublet chemotherapy has been the main initial treatment for extensive-stage small cell lung cancer (ES-SCLC). While the initial response rate is relatively high, approximately 80%, patient survival outcomes remain suboptimal. Clinical data indicate that the median survival time typically does not exceed 12 months, and fewer than 5% of patients survive beyond 24 months (6, 7). Since 2019, following the groundbreaking advancements of immune checkpoint inhibitors in oncology and based on the results of two pivotal clinical trials, IMpower133 and CASPIAN, the combination of immune checkpoint inhibitors (ICIs) (e.g., atezolizumab and durvalumab) with chemotherapy has emerged as the first-line standard treatment for ES-SCLC (8, 9). These studies show that combining immune checkpoint inhibitors with standard chemotherapy significantly prolongs overall survival (OS) and progression-free survival (PFS) in ES-SCLC patients, with median survival exceeding 12 months for the first time (8, 10). Immune checkpoint inhibitors have received FDA approval for both first-line and third-line treatment of extensive-stage or recurrent SCLC (11).

Immunotherapy for ES-SCLC still faces many challenges. Notably, the overall response rate (ORR) of SCLC to immunotherapy is lower compared to non-small cell lung cancer (NSCLC), and the proportion of patients deriving clinical benefit is limited. Specifically, only approximately 30% of SCLC patients achieve meaningful responses to immunotherapy (12). PD-L1 expression and TMB are potential biomarkers for predicting immune therapy response in SCLC patients (13). However, the predictive value of PD-L1 expression in SCLC remains to be fully validated, as research indicates that PD-L1 expression in SCLC patients is typically low and its correlation with the efficacy of ICIs has not been fully elucidated (14, 15). While TMB has been found to be positively correlated with the response to immunotherapy in some studies, its clinical utility is constrained by inconsistent detection methods and varying threshold standards (16). There is a pressing demand for clinically significant biomarkers to improve the rational application of ICIs in ES-SCLC therapy.

The immune checkpoint pathways not only mediate cellular interactions within the tumor microenvironment but also regulate systemic immune responses in circulation. These responses include changes in peripheral blood parameters, which are closely associated with the efficacy of immunotherapy. Evidence has highlighted the critical role of inflammatory responses in tumor initiation, progression, and immune evasion mechanisms (17, 18). In SCLC, the inflammatory process is considered a critical factor contributing to tumor cell proliferation and metastasis, thereby driving tumor progression via the activation of multiple oncogenic signaling pathways (19). Previous studies have demonstrated that peripheral blood biomarkers, including the neutrophil-to-lymphocyte ratio (NLR) (20), the lymphocyte-to-monocyte ratio (LMR) (21), the prognostic nutritional index (PNI) (22), and the platelet-to-lymphocyte ratio (PLR) (23), not only reflect systemic inflammatory states but are also significantly associated with the prognosis of various cancers, particularly SCLC. In addition, the lung immune prognostic index (LIPI), introduced by Mezquita et al. in 2018 (24) is a composite scoring system derived from baseline levels of the derived neutrophil-to-lymphocyte ratio (dNLR) and lactate dehydrogenase (LDH). Based on these parameters, LIPI categorizes patients into three risk groups: grade 0 (low-risk), grade 1 (intermediate-risk), and grade 2 (high-risk). LIPI has been utilized to assess the efficacy of immune checkpoint inhibitors (ICIs) and predict treatment responses across a range of solid tumors (24, 25). However, among ES-SCLC patients receiving ICIs therapy, the predictive performance of LIPI has yielded inconsistent results. Consequently, it remains uncertain whether LIPI can reliably predict the prognosis of ES-SCLC patients undergoing ICIs treatment.

Therefore, this meta-analysis seeks to consolidate current data to clarify the prognostic impact of baseline LIPI, as well as its component indicators (LDH and dNLR levels), on patients with ES-SCLC receiving ICIs therapy. A comprehensive meta-analysis of these three parameters will provide a more precise evaluation of LIPI’s predictive capability, potentially aiding in the optimization of patient stratification strategies, identification of subgroups likely to benefit from ICIs treatment, and ultimately improving both therapeutic outcomes and long-term survival for ES-SCLC patients.

## 2 Materials and methods

### 2.1 Protocol and guideline

This meta-analysis was performed adhering to the PRISMA 2020 guidelines for systematic reviews and meta-analyses (26). The registration number for this study on the PROSPERO platform is: CRD420251123579.

### 2.2 Literature search strategy

We performed a comprehensive search in the PubMed, Web of Science, Cochrane Library, and Embase databases for published studies up to January 1, 2025. To minimize potential omissions, we implemented a broad search strategy (**Supplementary Table S1** of **Supplementary File 1**). Articles considered for inclusion in this study were independently screened by two authors based on their titles and abstracts, followed by the downloading of full texts for relevant papers. Any disagreements between the two reviewers during the study-selection process were resolved through discussion and consensus. If consensus could not be reached, a third reviewer was consulted to make the final decision.

### 2.3 The criteria for inclusion and exclusion criteria

This study included research that met the following criteria: 1) Patients were diagnosed with ES-SCLC based on pathological and radiological evidence (27); 2) Patients underwent ICIs therapy, either alone or alongside chemotherapy or other treatments; 3) Evaluate the correlation between LIPI score assessed based on pre-immunotherapy dNLR value and LDH level, and the efficacy of immunotherapy; 4) Outcome measures for immunotherapy were defined as OS and PFS; 5) Articles provided hazard ratio (HR) and 95% confidence interval (95% CI) for OS and PFS; 6) Studies were observational in nature, including both prospective and retrospective designs.

This study excluded research meeting the following criteria: 1) Duplicate publications; 2) Comments, errata, or review articles reporting only other indicators or effects; 3) Studies failing to report the relationship between LIPI, LDH, dNLR, and PFS or OS in ES-SCLC patients treated with ICIs; 4) studies lacking significant outcome effects; 5) low-quality studies; 6) studies with inadequate data. 7) grey literature.

### 2.4 Evaluation of quality and extraction of data

This meta-analysis strictly followed the PRISMA 2020 guidelines (**Supplementary Table S9**) (26). Two authors independently reviewed the literature based on predefined criteria and evaluated its quality using the Newcastle-Ottawa Scale (NOS). In cases of disagreement, a third author was consulted for arbitration. The quality of studies based on NOS scores was categorized as follows: NOS scores ≥ 7 indicate high quality, scores ranging from 5 to < 7 as medium quality, and scores < 5 as low quality (28). Only studies with NOS scores ≥ 6, considered to be of acceptable high quality, were included in this analysis. The following data were extracted from the included studies: article title, first author’s name, publication year, country of study, study design (retrospective or prospective), sample size, disease stage (extensive or limited), study duration, age distribution, gender proportion, specific ICIs agents used, threshold values and LIPI comparisons, cutoff values for LDH and dNLR, study endpoints, HR values, and 95% CI.

### 2.5 The selection of estimate effect and 95% CI

In studies examining the impact of baseline LIPI and its component indicators (LDH and dNLR) on OS or PFS, when both univariate and multivariate analyses are performed, the results of multivariate analysis are typically preferred. This is because multivariate analysis accounts for potential confounding factors, providing a more robust estimation of the associations. Furthermore, some studies categorized baseline LIPI, LDH, and dNLR using specific cutoff values; however, the observed trends across these studies were inconsistent. Consequently, we performed normalization of the estimates and 95% confidence intervals derived from studies utilizing cutoff values to ensure comparability (29). For LIPI, we recoded “poor” as “2”, “intermediate” as “1”, and “good” as “0”, and consistently reformatted the comparison from “0 vs. 1 vs. 2” to “2 vs. 1 vs. 0”. For LDH and dNLR, we standardized the comparison by reordering “Low vs. high” to “High vs. low”.

### 2.6 Statistical analysis

Statistical analyses were performed using R software (version 4.4.3, R Foundation for Statistical Computing, Vienna, Austria). We constructed forest plots and summarized the hazard ratios along with their corresponding 95% CI to estimate the overall effects of LIPI, LDH, and dNLR on OS and PFS. The extent of heterogeneity across studies was assessed using the I² statistic. I² of 25%, 50%, and 75% represent low, medium, and high heterogeneity, respectively. Significant heterogeneity is indicated by an I² value over 50% or a P value below 0.05, warranting the use of a random-effects model (30). A fixed-effects model is applied when heterogeneity lacks statistical significance. If discrepancies are observed between the results of the two models, the random-effects model should be prioritized, as it is more conservative and robust in accounting for variations in population and treatment characteristics (31). Publication bias among the included studies was assessed using funnel plots and Begg’s test (32). Subgroup analyses explored potential heterogeneity sources, and sensitivity analyses assessed the robustness of pooled results by sequentially excluding individual studies. The significance level was set at α = 0.05.

## 3 Results

### 3.1 Literature search and selection

**Figure 1:**
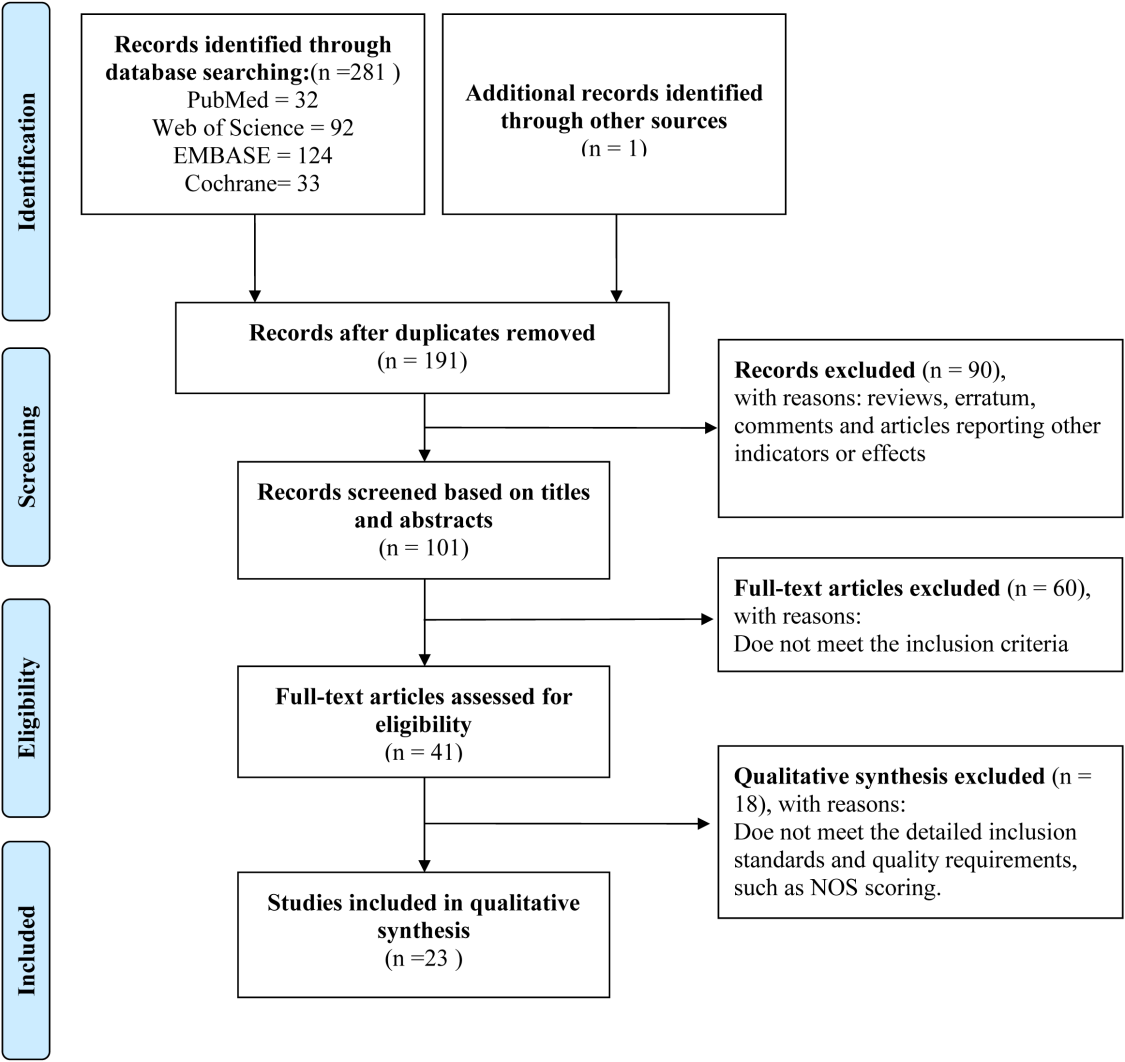
Flowchart showing the screening process for included articles.

The investigation employed a structured literature retrieval strategy across four core biomedical databases (PubMed, Web of Science, EMBASE, and Cochrane Library), combining controlled vocabulary terms with keyword searches. This approach yielded 281 potentially relevant citations before initiating the deduplication process. Specifically, 32 records were retrieved from PubMed, 92 from Web of Science, 124 from EMBASE, and 33 from Cochrane Library. Additionally, one relevant article was identified through other sources. After duplicates were removed using EndNote software, 191 independent studies were retained for the initial screening phase. Based on their titles and abstracts, 90 studies were excluded from the 191 studies. The full texts of the remaining 101 studies were retrieved for detailed evaluation. Following the application of the predefined inclusion and exclusion criteria, 41 studies were initially selected; subsequently, 18 were excluded after further assessment, leaving 23 high-quality studies for qualitative synthesis (**Figure 1**, **Table 1**) (33–55).

**Table 1:** Basic characteristics of LIPI, LDH and dNLR included studies.

Author	Published date	Country	Sample size	Age	Male/Female	Study type	ICIs	Threshold and comparison of LIPI/Cut off value of LDH/Cut off value of dNLR	Endpoint	NOS
Laura Bonanno et al. (54)	2024	Italian	89	69(Media)[61,75]	1.56	R	Atezolizumab	LIPI:0: dNLR ≤ 3 and LDH≤ULN;1:dNLR>3 or LDH>ULN; 2: dNLR>3 and LDH>ULN; 2 vs. 0	PFS, OS	8
Wei-Xiang Qi et al. (42)	2021	United States and Europe	53	≥65(49.1%,65(Media)	1.79	P	Atezolizumab	LIPI:0: dNLR ≤ 3 and LDH≤ULN; 1: dNLR>3 or LDH>ULN; 2: dNLR>3 and LDH>ULN; 2 vs. 0 and 1 vs. 0	OS	9
Lingling Li et al. (45)	2021	China	100	60(Media)≥60(52%)	7.33	R	Nivolumab, pembrolizumab, sintilimab	LIPI:0: dNLR<4 and LDH<283; 1:(dNLR<4 and LDH≥283 U/L) or (dNLR≥4.0 and LDH < 283 U/L); 1/2 vs. 0	PFS, OS	6
Ying Yi et al. (36)	2022	China	65	58(Media)	4.42	R	NR	LIPI:0: dNLR<4 and LDH<283;1:(dNLR<4 and LDH≥283 U/L) or (dNLR≥4.0 and LDH < 283 U/L); 1/2 vs. 0 LDH:>245U/L dNLR:>3U/L	PFS	6
Junjie Dang et al. (53)	2024	China	113	≥65(69%)average:61	2.23	R	PD-1/PD-L1 inhibitor	LIPI:0: dNLR ≤ 3 and LDH≤ULN; 1: dNLR>3 or LDH>ULN; 2: dNLR>3 and LDH>ULN; 2 vs. 0 and 1 vs. 0 LDH:≥ 146.5U/L	OS	7
Kana Hashimoto et al. (50)	2024	Japan	228	70(Media)	2.68	R	Durvarumab, atezolizumab	LIPI:0: dNLR ≤ 3 and LDH ≤ 260; 1: dNLR>3 or LDH>260; 2: dNLR>3 and LDH>260; 2 (vs. 0 and 1) LDH:260U/L	OS	7
L. Mezquita et al. (43)	2018	Europe	66	63(Media)	4.00	R	NR	LIPI: NR; 2 vs. 0	PFS, OS	6
Meiling Zhang et al. (34)	2024	China	120	>60(49.2%)	1.61	R	PD-1/PD-L1 inhibitor	LIPI:0: dNLR ≤ 3 and LDH≤ULN; 1: dNLR>3 or LDH>ULN; 2: dNLR>3 and LDH>ULN; 2 vs. 1 vs. 0	PFS, OS	9
Jie Zhao et al. (33)	2023	China	341	≥65(43.1%)average:62 ± 8.7	9.03	R	Durvarumab, atezolizumab	LIPI: NR; 2 vs. 0 and 1 vs. 0	PFS, OS	8
Jingyuan Xie et al. (37)	2024	China	116	≥65(56.90%)	10.6	R	PD-1/PD-L1 inhibitor	LIPI: NR; 2 vs. 0 and 1 vs. 0 LDH:>245U/L	PFS, OS	8
Yang Wang et al. (38)	2023	Australia	75	68.7(Media), ≥65(44%)	2.13	R	Atezolizumab	LDH: NR	PFS, OS	8
Shira Sagie et al. (41)	2022	Israel	54	67[63,71](Media)	1.35	R	Atezolizumab, durvalumab	LDH:>350U/L	OS	8
Jeong Uk Lim et al. (44)	2022	Korea	41	69(Media)	19.5	R	Atezolizumab	LDH: NR dNLR: NR	PFS, OS	9
Seoyoung Lee et al. (46)	2022	Korea	68	68(Media)	8.71	R	Atezolizumab	LDH: NR	PFS, OS	9
Ran Zeng et al. (35)	2021	China	53	≥65(49.10%)	1.79	R	Atezolizumab	LDH: NR	PFS, OS	6
Ran Zeng et al. (35)	2021	China	84	≥65(48.8%)	1.8	R	PD-1/PD-L1 inhibitor	LDH: NR	PFS, OS	6
Jinfeng Guo et al. (51)	2023	Project Data Sphere platform	53	≥65(49.10%)	1.79	P	Atezolizumab	LDH: NR dNLR:≥1.79U/L	PFS, OS	7
Zhanpeng Kuang et al. (47)	2024	United States	129	64(Media)	1.29	R	Pembrolizumab, nivolumab, cemiplimab, atezolizumab, durvalumab, avelumab, ipilimumab	LDH:>190U/L dNLR:>2.5U/L	OS	9
Jong-Min Baek et al. (55)	2024	Korea	55	72(Media)[66,77],≥70(58.2%)	17.33	R	Atezolizumab	LDH:>250U/L	PFS, OS	6
Ping-Chih Hsu et al. (49)	2024	China	72	65(Media)	23	R	Atezolizumab, durvalumab	LDH:>260U/L	OS	7
Yuxin Jiang et al. (48)	2024	China	118	64 ± 8(average)	5.94	R	PD-1/PD-L1 inhibitor	LDH:>259U/L	OS	8
Ruiting Song et al. (40)	2024	China	231	61.2 ± 8.2(average)	3.81	R	Durvalumab, atezolizumab	LDH:>273U/L	PFS, OS	7
Bingbing Wang et al. (39)	2024	China	213	61(Media), ≥61(51.8%)	2.7	R	NR	LDH:≥ 236U/L	OS	7
Julia Grambow -Velilla et al. (52)	2023	France	36	67(Media)	2.27	R	Atezolizumab, durvalumab	dNLR:>3U/L	PFS, OS	6

NR, not reported; P, Prospective; R, retrospective; ICIs, immune checkpoint inhibitors; LIPI, lung immune prognostic index; NOS, Newcastle-Ottawa Scale; PD-1, programmed death-1; PDL1, programmed cell death 1 ligand 1; dNLR, derived neutrophil-to-lymphocyte ratio; ULN, upper limit of normal level; LDH, lactate dehydrogenase; OS, overall survival; PFS, progression free survival.

### 3.2 The basic characteristics included in the study

A total of 1,291 participants were included in 10 studies published between 2018 and 2024 that focused on the lung immune prognostic index. Among these, seven studies evaluated PFS as the clinical outcome, nine studies assessed OS, and six studies reported both PFS and OS. Among these studies, the majority were retrospective in nature, with only one study being prospective. All studies utilized the dNLR and LDH levels for risk stratification. The LIPI was classified into three risk levels: 0 for low risk, 1 for intermediate risk, and 2 for high risk. And a total of 1,768 participants were included in 17 studies on LDH published between 2021 and 2024. Among these studies, 10 evaluated PFS as the clinical outcome, 16 assessed OS as the clinical outcome, and 9 reported both PFS and OS. One study was prospective, while the remaining 16 were retrospective. Different cutoff values were utilized to categorize LDH levels across the studies, with six of them not reporting specific cutoff values. About dNLR, a total of 324 participants were included in 5 studies published between 2022 and 2024 focusing on dNLR. Among these, four studies evaluated PFS as the clinical outcome, four assessed OS, and three reported both PFS and OS. One study was prospective, while the remaining four were retrospective. Among these studies, four utilized different cutoff values to stratify the risk based on dNLR levels, while one study did not specify the exact cutoff value. The quality of the included studies was assessed using the Newcastle-Ottawa Scale, with scores between 6 and 9 points. Studies with an NOS score of ≥ 6 were considered eligible for inclusion (**Supplementary Table S10**). **Table 1** provides a summary of the characteristics of the included studies.

### 3.3 Estimated values of the combined effect and 95% CI

We assessed the influence of baseline LIPI, LDH, and dNLR levels on OS and PFS in ES-SCLC patients receiving ICIs, based on distinct clinical outcomes.

**Figure 2:**
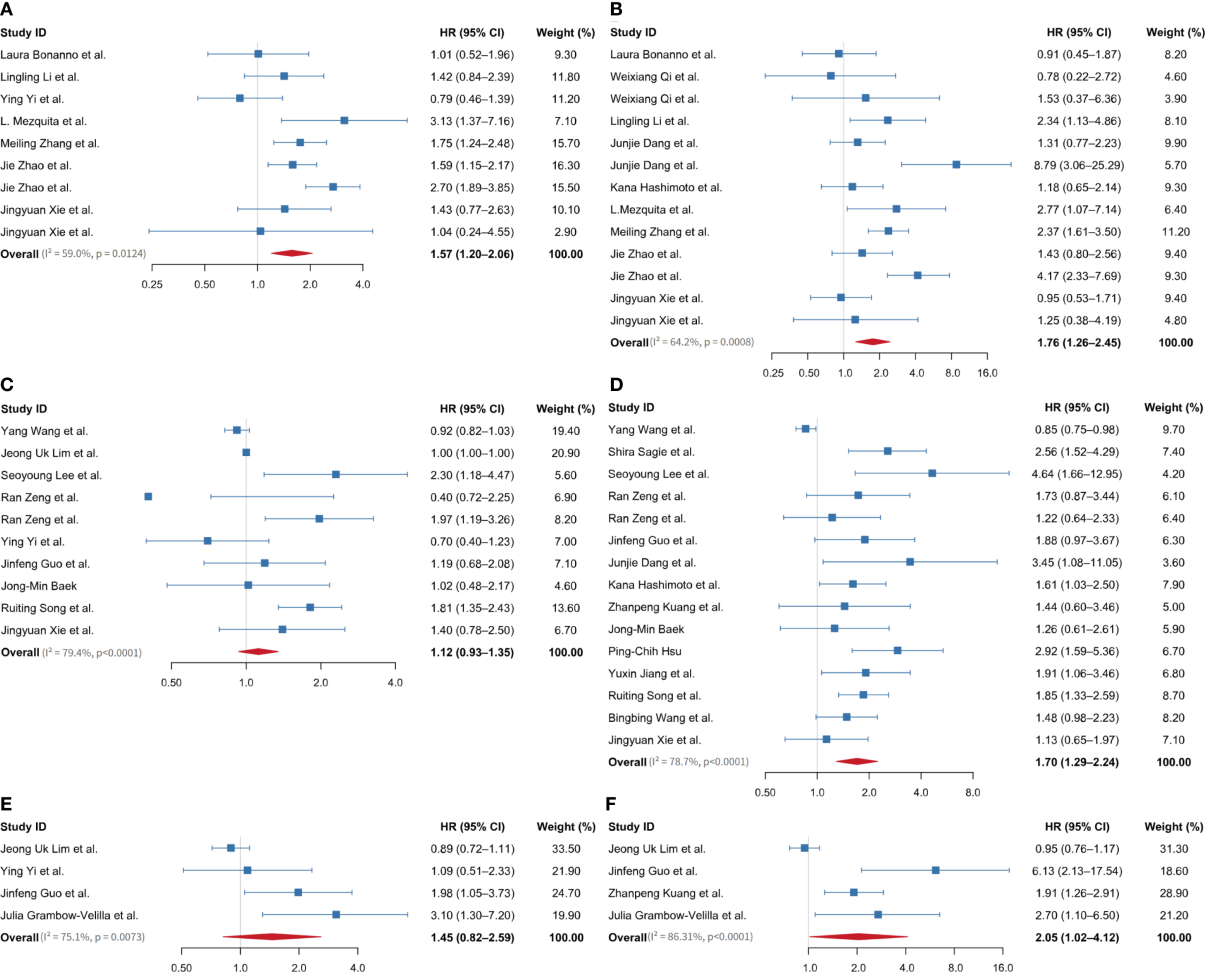
Forest plot showing overall HR and 95% CI between baseline LIPI, LDH, and dNLR levels on prognosis of extensive-stage small cell lung cancer patients treated with immune checkpoint inhibitors. **(A)** between LIPI and PFS; **(B)** between LIPI and OS; **(C)** between LDH and PFS; **(D)** between LDH and OS; **(E)** between dNLR and PFS; **(F)** between dNLR and OS. LIPI, lung immune prognostic index; LDH, lactate dehydrogenase; dNLR, derived neutrophil-to-lymphocyte ratio; HR, hazard ratio; PFS, progression-free survival; OS, overall survival.

**Figure 2A**, **Supplementary Table S2** in **Supplementary File 1** illustrate that nine studies examined the link between baseline LIPI and PFS in ES-SCLC patients receiving ICIs. The pooled analysis indicated that a higher LIPI correlated with reduced PFS, showing a combined effect size of 1.57 (95% CI 1.20-2.06), I² = 59.0%, P = 0.013. Furthermore, data from 13 studies examined the relationship between baseline LIPI and OS in ES-SCLC patients treated with ICIs. The pooled analysis indicated a significant association between elevated LIPI and reduced OS, with a combined effect size of 1.76 (95% CI 1.26-2.45), I² = 64.2%, P< 0.001 (**Figure 2B**, **Supplementary Table S3** in **Supplementary File 1**).

Ten studies examined the link between baseline LDH levels and PFS in ES-SCLC patients receiving ICIs. The pooled effect size was 1.12 (95% CI 0.93-1.35), I² = 79.4%, P < 0.001 (**Figure 2C**, **Supplementary Table S4** in **Supplementary File 1**). Seventeen studies examined the link between baseline LDH levels and OS in ES-SCLC patients receiving ICIs, resulting in a pooled effect size of 1.0 (95% CI 0.99-1.01) (**Supplementary Figure S1A**). A sensitivity analysis, conducted by sequentially omitting one study at a time, showed that excluding two related studies from a single article significantly impacted the overall pooled effect value (**Supplementary Figure S1B**). After excluding this article, 15 studies were retained for analysis. The pooled results demonstrated that elevated LDH levels were significantly associated with poorer OS, with a pooled effect size of 1.70 (95% CI 1.29-2.24), I² = 78.7%, P < 0.001 (**Figure 2D**, **Supplementary Table S5** in **Supplementary File 1**).

A total of four studies investigated the associations between baseline dNLR and both PFS and OS in ES-SCLC patients treated with ICIs. The pooled effect size for PFS was 1.45 (95% CI 0.82-2.59), I² = 75.1%, P = 0.007 (**Figure 2E**, **Supplementary Table S6** in **Supplementary File 1**). For OS, the pooled effect size was 2.05 (95% CI 1.02-4.12), I² = 86.31%, P < 0.001 (**Figure 2F**, **Supplementary Table S7** in **Supplementary File 1**).

### 3.4 Subgroup analysis

To systematically assess the impact of LIPI and its components, LDH and dNLR, on the survival outcomes of ES-SCLC patients treated with ICIs, we performed a subgroup analysis. Given that all pooled effect estimates exhibited significant heterogeneity (I² > 50%), this analysis aimed to deepen our understanding of the prognostic significance of LIPI and its components in these patient populations and to explore potential sources of heterogeneity. The primary focus was on key factors including average age, univariate and multivariate analyses, critical thresholds, comparison approaches, cutoff values, population characteristics, publication dates, differences between Eastern and Western populations, and gender distribution.

**Figure 3:**
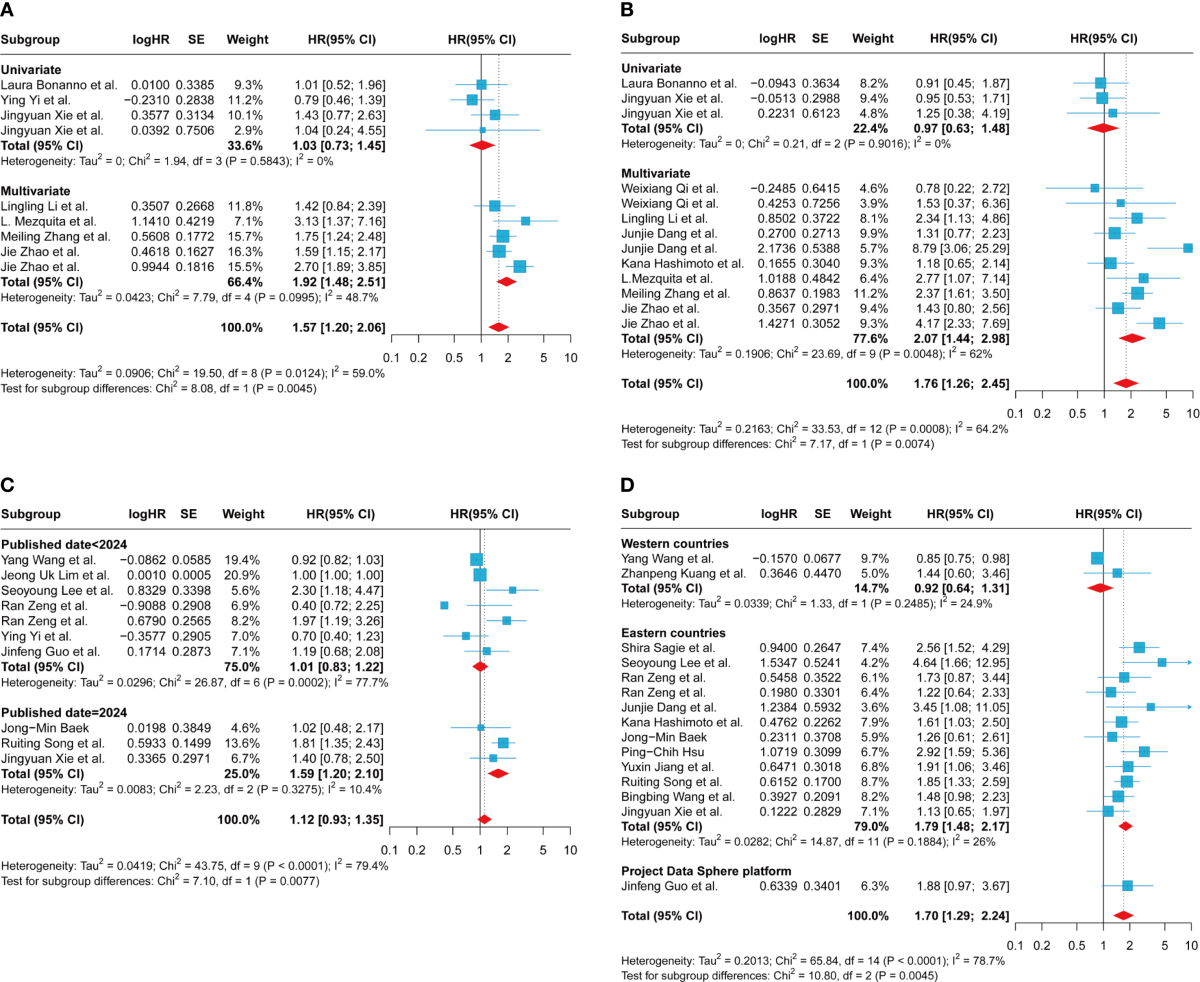
Subgroup analysis of the association between LIPI, LDH and progression-free survival and overall survival in patients with extensive-stage small cell lung cancer treated with immune checkpoint inhibitors. **(A)** between LIPI and progression-free survival based on univariate or multivariate; **(B)** between LIPI and overall survival based on univariate or multivariate; **(C)** between LDH and progression-free survival based on published date; **(D)** between LDH and overall survival based on country. LIPI, lung immune prognostic index; LDH, lactate dehydrogenase.

The subgroup analysis of LIPI on the clinical outcome PFS revealed that studies grouped by univariate and multivariate analyses exhibited reduced heterogeneity compared to the overall group. The univariate analysis subgroup revealed I² = 0%, P = 0.5843, with a pooled effect estimate of 1.03 (95% CI 0.73-1.45). In contrast, the multivariate analysis subgroup showed I² = 48.7%, P = 0.10, with a pooled effect estimate of 1.57 (95% CI 1.20-2.06) (**Figure 3A**). These findings suggest that the grouping method (univariate vs. multivariate) may be a potential source of heterogeneity. In studies evaluating LIPI’s effect on PFS, grouped by publication date post-2024, heterogeneity was minimal (I² = 0%, P = 0.50), with a pooled effect estimate of 1.51 (95% CI 1.15-1.98) (**Supplementary Figure S2A**). Studies grouped by the 1 vs. 0 comparison exhibited reduced heterogeneity (I² = 0%, P = 0.768), with a pooled effect estimate of 1.57 (95% CI 1.17-2.06) (**Supplementary Figure S2B**). Additionally, studies with an average age > 62 years showed less heterogeneity compared to those with an average age ≤ 62 years (I² = 0%, P = 0.50), with a pooled effect estimate of 1.51 (95% CI 1.15-1.98) (**Supplementary Figure S2C**). Finally, studies including populations ≤ 116 demonstrated reduced heterogeneity (I² = 38.7%, P = 0.148), with a pooled effect estimate of 1.57 (95% CI 1.17-2.06) (**Supplementary Figure S2D**). However, subgroup analyses based on gender ratio and regional differences failed to identify significant sources of heterogeneity (**Supplementary Figures S2E, F**). The aforementioned subgroup analyses demonstrated that a high LIPI was significantly associated with poorer PFS across groups stratified by statistical methods (univariate and multivariate analyses), average age, comparison methods, population size, and publication date.

The subgroup analysis of LIPI on the clinical outcome OS revealed that in the univariate subgroup, lower heterogeneity was observed (I² = 0%, P = 0.902), with a pooled effect estimate of 0.97 (95% CI 0.63-1.48) (**Figure 3B**). In studies published before 2023, heterogeneity was minimal (I² = 0%, P = 0.405), with a combined effect estimate of 1.97 (95% CI 1.20-3.22) (**Supplementary Figure S3A**). Subgroup analyses using the 1 vs. 0 grouping method demonstrated minimal heterogeneity (I² = 0%, P = 0.681) and yielded a pooled effect estimate of 1.18 (95% CI 0.86-1.62) (**Supplementary Figure S3B**). Studies stratified by an average age > 63 years showed minimal heterogeneity (I² = 0%, P = 0.965), with a pooled effect estimate of 1.04 (95% CI 0.75-1.43) (**Supplementary Figure S3C**). Finally, in subgroup analyses based on country and region, the group of Western developed countries demonstrated moderate heterogeneity (I² = 26.4%, P = 0.253). The pooled effect estimate was 1.30 (95% CI 0.72 - 2.35) (**Supplementary Figure S3D**). However, subgroup analyses based on the included population and gender ratio failed to reveal significant sources of heterogeneity (**Supplementary Figures S3E, F**). Subgroup analyses confirmed that a higher LIPI was significantly linked to reduced OS.

The subgroup analysis aimed to assess the effect of the LIPI component, LDH, on the clinical outcome PFS. This analysis revealed that studies published before and after 2024, using 2024 as the cutoff year for grouping, exhibited different levels of heterogeneity. Specifically, studies published in or before 2024 demonstrated reduced heterogeneity (I² = 10.4%, P = 0.328), with a combined effect estimate of 1.59 (95% CI 1.20-2.10) (**Figure 3C**). Additionally, subgroup analyses based on geographic region showed that studies from Western developed countries had lower heterogeneity compared to those from Eastern countries (I² = 0%, P = 0.380), with a combined effect estimate of 0.93(95% CI 0.83-1.04) (**Supplementary Figure S4A**). However, subgroup analyses based on other factors, including the cutoff value, gender ratio, and average age, failed to reveal significant sources of heterogeneity (**Supplementary Figure S4B–F**).

The subgroup analysis of the LIPI component LDH on the clinical outcome of OS revealed that, in studies stratified by country or region, both subgroups exhibited relatively low heterogeneity. Specifically, one subgroup showed an I² of 24.9%, P = 0.249, with a pooled effect estimate of 0.92 (95% CI 0.64–1.31), while the other subgroup had an I² of 26%, P = 0.189, with a pooled effect estimate of 1.79 (95% CI 1.48–2.17) (**Figure 3D**). These findings suggest that differences in study publication locations across countries or regions may potentially contribute to observed heterogeneity. It is worth noting that the single-factor analysis group exhibited significantly lower heterogeneity compared to the multi-factor analysis group (I² = 0%, P = 0.567), with a pooled effect estimate of 1.60 (95% CI 1.26-2.02) (**Supplementary Figure S5A**). Similarly, the subgroup with a population over 84 years old demonstrated reduced heterogeneity relative to the subgroup with a population aged 84 years or younger (I² = 0%, P = 0.624), with a pooled effect estimate of 1.64 (95% CI 1.36-1.98) (**Supplementary Figure S5B**). The subgroup with an average age ≤ 67 years exhibited lower heterogeneity compared to the subgroup with an average age > 67 years (I² = 13.7%, P = 0.320), with a pooled effect estimate of 1.85 (95% CI 1.52-2.25) (**Supplementary Figure S5C**). Similarly, the subgroup with a sex ratio ≥ 2.68 demonstrated reduced heterogeneity relative to the subgroup with a sex ratio ≤ 2.68 (I² = 32.1%, P = 0.171), with a pooled effect estimate of 1.74 (95% CI 1.39-2.19) (**Supplementary Figure S5D**). Groups with clearly defined cutoff values exhibited significantly lower heterogeneity compared to those without (I² = 13.7%, P = 0.317), with a pooled effect estimate of 1.77 (95% CI 1.47-2.12) (**Supplementary Figure S5E**). Additionally, when the publication year of 2024 was used as the cutoff value, studies published after 2024 demonstrated reduced heterogeneity (I² = 2.9%, P = 0.411), with a pooled effect estimate of 1.69 (95% CI 1.41-2.02) (**Supplementary Figure S5F**). Subgroup analysis stratified by population size and gender ratio revealed that the results of each subgroup were consistent with the overall pooled effect estimate (**Figure 2D**). These findings suggest that elevated LDH levels are associated with worse OS.

### 3.5 Publication bias and sensitivity analysis

**Figure 4:**
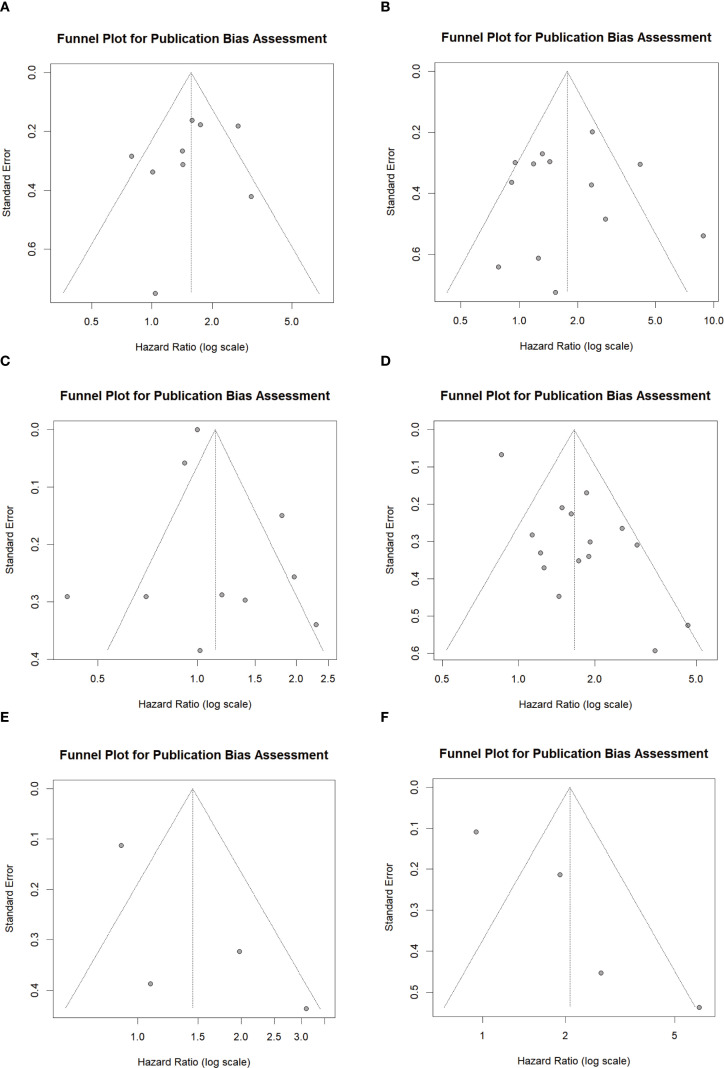
Funnel plot showing the publication bias. **(A)** between LIPI and PFS; **(B)** between LIPI and OS; **(C)** between LDH and PFS; **(D)** between LDH and OS; **(E)** between dNLR and PFS; **(F)** between dNLR and OS. LIPI, lung immune prognostic index; LDH, lactate dehydrogenase; dNLR, derived neutrophil-to-lymphocyte ratio; PFS, progression-free survival; OS, overall survival.

In this meta-analysis, funnel plots and Begg’s test were employed to assess potential publication bias. For the clinical outcomes of LIPI on PFS and OS, the funnel plots exhibited symmetry (**Figures 4A, B**). The Begg’s test results for PFS and OS were z = -0.83 (P = 0.404) and z = 0.49 (P = 0.626), respectively (**Supplementary Table S6** in **Supplementary File 1**).The funnel plots for LDH in relation to PFS and OS exhibited symmetry (**Figures 4C, D**), with Begg’s test results of z = -0.09 (P = 0.929) for PFS and z = 0.25 (P = 0.805) for OS (**Supplementary Table S7** in **Supplementary File 1**). Similarly, the funnel plots for dNLR regarding PFS and OS also demonstrated symmetry (**Figures 4E, F**), with Begg’s test values of z = 1.36 (P = 0.174) for both PFS and OS (**Supplementary Table S7** in **Supplementary File 1**). The above results suggest that no significant publication bias exists in the studies included in this meta-analysis for LIPI and its component indicators, LDH and dNLR.

**Figure 5:**
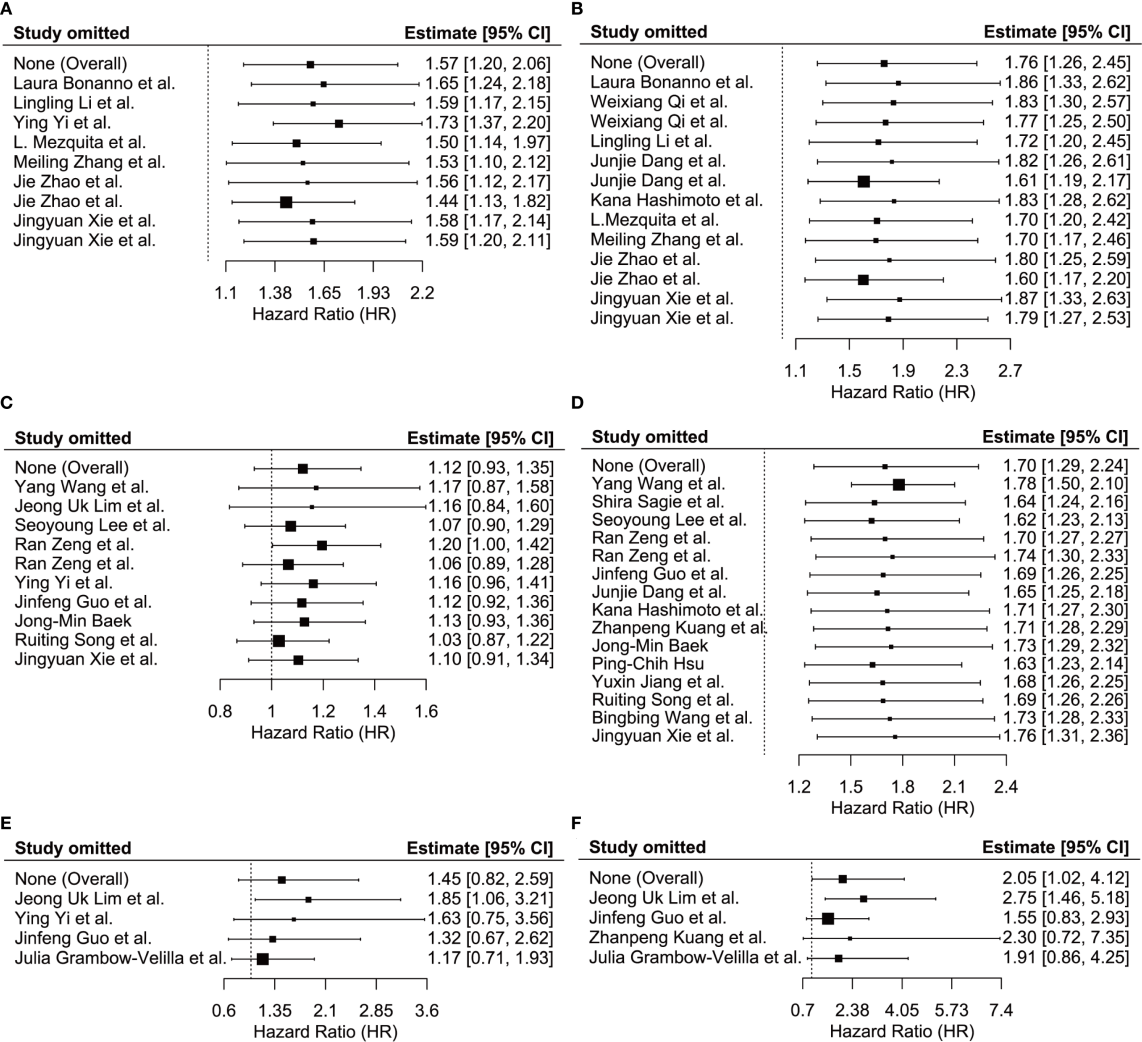
Sensitivity analysis showed the stability of the results. **(A)** between LIPI and PFS; **(B)** between LIPI and OS; **(C)** between LDH and PFS; **(D)** between LDH and OS; **(E)** between dNLR and PFS; **(F)** between dNLR and OS. LIPI, lung immune prognostic index; LDH, lactate dehydrogenase; dNLR, derived neutrophil-to-lymphocyte ratio; PFS, progression-free survival; OS, overall survival.

We conducted a sensitivity analysis to assess the robustness of the relationship between LIPI, LDH, dNLR, and OS/PFS by leave-one-out sensitivity analysis to determine if this significantly impacted the pooled estimates in our meta-analysis. None of the included studies significantly influenced the relationships between LIPI and PFS (**Figure 5A**), LIPI and OS (**Figure 5B**), LDH and PFS (**Figure 5C**), LDH and OS (**Figure 5D**), dNLR and PFS (**Figure 5E**), or dNLR and OS (**Figure 5F**) in patients with ES-SCLC undergoing immunotherapy. These findings suggest that the pooled estimates of this meta-analysis are robust.

## 4 Discussion

This meta-analysis systematically assessed the prognostic predictive value of the baseline LIPI and its components, LDH and dNLR, in ES-SCLC patients receiving ICIs. The pooled analysis revealed that LIPI exhibited robust predictive capability. Higher LIPI levels were notably linked to poorer PFS and OS, with combined effect sizes of 1.57 (95% CI 1.20–2.06) and 1.76 (95% CI 1.26–2.45). These findings indicate that the LIPI, a straightforward inflammatory marker derived from routine laboratory parameters, has potential clinical utility. It may be useful for patient stratification and predicting treatment efficacy in SCLC immunotherapy. The pooled analysis of LDH revealed that elevated LDH levels were significantly associated with OS, with a HR of 1.70 (95% CI 1.29–2.24). In contrast, the impact of LDH on PFS did not reach statistical significance (HR = 1.12, 95% CI 0.93–1.35), indicating that its utility in reflecting disease progression may be limited. As another inflammation-related biomarker contributing to LIPI, dNLR exhibited a certain trend in this study. The results showed that while elevated dNLR levels were not significantly associated with PFS (HR = 1.45, 95% CI 0.82 - 2.59), they demonstrated a more pronounced predictive effect on OS (HR = 2.05, 95% CI 1.02 - 4.12, I² = 86.31%). This suggests that dNLR may be better suited for evaluating long-term survival outcomes. In conclusion, the meta-analysis results of LDH and dNLR further reinforce, to some extent, the significant predictive value of LIPI—a composite scoring system based on LDH and dNLR—in the immunotherapy of ES-SCLC.

Inflammation is widely acknowledged as a critical factor that significantly contributes to the initiation and progression of cancer (56). The heterogeneity of the tumor microenvironment is significantly associated with the prognosis of various tumors (57, 58), while inflammation can promote angiogenesis, tumor proliferation, and metastasis, activate fibroblasts, and disrupt adaptive immune responses, making it a key component of the tumor microenvironment (59, 60).The extent of inflammation is strongly associated with the prognosis of malignant tumors. It influences patient outcomes in cancer patients by inducing immune tolerance to tumor cells, facilitating tumor growth and metastasis, and activating oncogenic signaling pathways (61). Chronic inflammation drives tumor angiogenesis, immune escape, and matrix remodeling by activating M2 macrophages, regulatory T cells, and myeloid-derived suppressor cells, which in turn secrete pro-angiogenic and immunosuppressive factors. Additionally, it induces genomic instability, thereby accelerating carcinogenesis (62).Cancer-related inflammation drives tumor progression through the activation of local pro-inflammatory signals (e.g., inflammasome activation and cytokine release) and systemic inflammatory markers (e.g., elevated CRP levels and cachexia). This process facilitates angiogenesis, induces immunosuppression, and disrupts signaling pathways (e.g., STAT3 and NF-κB), thereby contributing to an aggressive tumor microenvironment (63). Currently, the development of blood-based biomarkers has gained significant momentum. Peripheral blood inflammatory parameters have demonstrated consistent prognostic value across various cancer types and clinical contexts, highlighting their potential utility in predicting the efficacy of cancer immunotherapy or monitoring tumor progression (20–23). Moreover, in contrast to the detection of biomarkers such as PD-L1 and TMB, routine blood sampling offers superior accessibility and enhances practicality in clinical applications. Compared with single peripheral blood indicators, the current trend involves integrating multiple parameters to improve the accuracy of prognostic prediction. For instance, the LIPI is a predictive scoring system that combines LDH levels and the dNLR (24).

Several studies have demonstrated that LIPI levels are correlated with the prognosis of various types of cancer. Additionally, LIPI has been shown to be significantly associated with both the treatment efficacy and survival outcomes in patients with clear cell renal cell carcinoma receiving tyrosine kinase inhibitors (TKIs). Patients in the low-risk group exhibited significantly longer median progression-free survival (mPFS) and OS compared to those in the high-risk group (64). LIPI is significantly associated with inferior survival outcomes in patients with metastatic renal cell carcinoma treated with ICIs or anti-angiogenic therapy (65). Research demonstrates that LIPI is a predictive factor for PFS and OS in patients with advanced non-small cell lung cancer treated with ICIs (24). LIPI is essential for assessing the prognosis of different tumors. Yi Wang et al.’s meta-analysis found that in NSCLC patients treated with ICIs, a higher LIPI was significantly associated with worse overall survival (HR = 2.50, 95% CI 2.09–2.99, p < 0.001) and progression-free survival (HR = 1.77, 95% CI 1.64–1.91, p < 0.001) (66). Wenquan Lu et al. conducted a meta-analysis. The study indicated that NSCLC patients treated with ICIs in the low-risk group showed notably extended OS and PFS (67). Additionally, the meta-analysis performed by Yusheng Guo et al. LIPI effectively stratifies prognosis for NSCLC and other solid tumors undergoing immune checkpoint inhibitor treatment (68). Numerous studies have explored the link between LIPI and the prognosis of tumor patients undergoing immune checkpoint inhibitor therapy. Meta-analyses and systematic reviews mainly concentrate on NSCLC, leaving limited evidence on the prognostic significance of LIPI in ES-SCLC patients undergoing ICIs treatment. Therefore, we performed this meta-analysis and systematic review to assess the prognostic predictive value of LIPI in ES-SCLC patients treated with ICIs. The aggregated results demonstrated that LIPI can act as a robust prognostic biomarker for this patient population. However, the specific mechanisms of action, the determination of optimal biomarker threshold values, and the differences in predictive efficacy among different treatment regimens still require in-depth exploration. In particular, more prospective studies are needed to validate the clinical utility of LIPI in ES-SCLC and to clarify its potential associations with tumor microenvironment characteristics as well as with treatment responsiveness. Furthermore, future research should also focus on strategies to combine LIPI with other biomarkers to improve the predictive accuracy of immunotherapy responses in patients with ES-SCLC.

The dNLR is calculated using the formula: neutrophil count/(white blood cell count - neutrophil count). Neutrophils promote angiogenesis via VEGF secretion, accelerate tumor proliferation by activating the PI3K pathway through elastase secretion, and suppress anti-tumor immune responses. Moreover, IL-17-positive T cells recruit neutrophils via CXC chemokines. The activation of the IL-17 signaling pathway is directly associated with resistance to ICIs, which further reinforces the link between elevated dNLR and immunotherapy resistance (69). Lymphocytes, conversely, suppress tumor progression via their cytotoxic activities and immune surveillance mechanisms (70, 71). Thus, the components of dNLR—the numerator (neutrophils) and denominator (white blood cells - neutrophils, i.e., lymphocytes + monocytes)—reflect the dynamic equilibrium between pro-tumorigenic and anti-tumorigenic forces within the tumor microenvironment (60, 72). The key distinction between dNLR and the classic neutrophil-to-lymphocyte ratio (NLR) is that NLR uses the lymphocyte count as its denominator, while dNLR uses the sum of lymphocytes and monocytes as its denominator (73). The dNLR allows for a more comprehensive reflection of the immune status of cancer patients. Lymphopenia and monocytosis are common characteristics in cancer; monocytes facilitate angiogenesis and immunosuppression by differentiating into tumor-associated macrophages (74). Emerging evidence indicates that dNLR is significantly linked to the prognosis of various tumors treated with immune checkpoint inhibitors, such as advanced melanoma (75), NSCLC (76), and metastatic renal cell carcinoma (77). Yan Ou et al. conducted a meta-analysis. Elevated dNLR levels were significantly linked to poorer OS and PFS in melanoma patients undergoing immune checkpoint inhibitor therapy (78). Additionally, the meta-analysis performed by Tao Yang et al. confirmed that a higher dNLR was a robust predictor of poorer OS and PFS outcomes in NSCLC patients treated with ICIs (79). Furthermore, the meta-analysis conducted by Shiqiang Su et al. revealed that an elevated dNLR prior to renal cell carcinoma treatment was significantly associated with reduced cancer-specific survival (CSS) and disease-free survival (DFS), but not OS. In prostate cancer, a higher dNLR correlated with poorer biochemical recurrence-free survival (BRFS) and OS. In urothelial carcinoma, an increased dNLR was linked to inferior OS and cancer-specific survival (CSS), though it did not affect disease-free survival (DFS) (80). However, current research lacks systematic reviews and meta-analyses regarding the treatment of ES-SCLC with ICIs. Our meta-analysis demonstrates that a higher dNLR fails to significantly predict PFS in ES-SCLC patients treated with ICIs, yet it exhibits a significant predictive value for OS. However, the conclusion may be associated with uncertainty in the pooled effect estimate due to the limited number of included studies and substantial heterogeneity among the four studies (OS: I² = 86.31%, PFS: I² = 75.1%). Additionally, the small sample size and the resultant insufficient power of funnel plots or Egger’s test may contribute to potential overestimation of the effect size. Therefore, this conclusion should be interpreted with caution. The unique neuroendocrine characteristics of SCLC may reshape the functions of neutrophils and monocytes; therefore, the mechanism underlying the association between SCLC and the dNLR needs to be clarified through further research on the tumor microenvironment. Additionally, future studies should include more well-designed trials and establish prospective validation cohorts to standardize aspects such as measurement methods and cutoff values of dNLR. This will help verify the accuracy of dNLR as a predictive biomarker for ICIs treatment in ES-SCLC, and to uncover the relevant mechanisms of its potential impact.

LDH, an enzyme prevalent in major human organs, facilitates the reversible conversion of lactic acid to pyruvic acid. Elevated serum LDH levels may serve as a biomarker reflecting cellular damage, inflammation, and necrosis (81). In tumors, the metabolic reprogramming of cancer cells is an important factor in tumor occurrence and development (82). LDH is considered a critical biomarker of metabolic reprogramming and proliferative activity. LDH levels directly correlate with overall tumor burden and invasiveness (83). Elevated LDH is closely linked to increased glycolytic activity and hypoxia-induced necrosis in tumors, which are typically associated with a significant tumor burden (84). Even under aerobic conditions, tumor cells predominantly utilize glycolysis for glucose metabolism. LDH catalyzes the transformation of pyruvate into lactate, thereby supporting the energy demands of rapid tumor growth (85). Additionally, the low pH induced by LDH-mediated acidification inhibits immune cell function and further enhances immunosuppression by promoting hypoxia. Hypoxia induces the activation of the hypoxia-inducible factor-1 (HIF-1), leading to the upregulation of vascular endothelial growth factor (VEGF), which facilitates abnormal angiogenesis and the formation of a dysfunctional tumor vasculature (86–89). These pathological changes collectively hinder immune cell infiltration and diminish the therapeutic efficacy of ICIs (87, 89). The prognostic significance of LDH levels has been evaluated in various cancers treated with immune checkpoint inhibitors. Xiaocui Liang et al.’s meta-analysis revealed that elevated LDH levels were significantly associated with poorer OS and PFS in patients with uveal melanoma receiving immune checkpoint inhibitor therapy (90). Additionally, Yongchao Zhang et al.’s meta-analysis indicated that high pretreatment LDH levels were correlated with inferior PFS and OS in melanoma patients undergoing immune checkpoint inhibitor treatment (91). Zhibo Zhang et al.’s meta-analysis demonstrated that elevated baseline LDH levels in patients with advanced non-small cell lung cancer receiving ICIs were significantly associated with shorter PFS and OS (92). Fausto Petrelli et al.’s meta-analysis indicated that elevated baseline LDH levels in melanoma patients treated with immunotherapy and BRAF inhibitors represented a poor prognostic factor (93). Despite the strong predictive value of LDH demonstrated in previous studies, systematic evaluations and meta-analyses on its prognostic role in ES-SCLC patients undergoing ICIs treatment are still lacking. Although our meta-analysis preliminarily confirmed that elevated LDH levels are significantly associated with poorer OS in ES-SCLC patients receiving ICIs treatment, it failed to demonstrate significant predictive value for PFS. This apparent contradiction, together with the unique biological characteristics of ES-SCLC, suggests that the role of LDH in ES-SCLC and its value as a predictor of ICIs efficacy may be specific. Therefore, more in-depth basic and clinical research is needed to reveal its potential mechanisms and clarify the details of its predictive value, such as determining the optimal cut-off value, exploring interactions with other factors, and assessing its practical utility in guiding clinical decision-making.

The study is subject to several limitations. First, all included studies were observational, mostly retrospective, with some conducted at single centers, which may introduce selection bias. Future research should address this limitation by confirming the findings through well-designed prospective cohort studies or randomized controlled trials. These studies can provide robust evidence to clarify the causal relationships between baseline LIPI, dNLR, and LDH levels and specific outcomes—such as survival or response rates—of SCLC patients treated with ICIs. Additionally, they serve to validate the findings of the current study. Secondly, the relatively small sample sizes in some included studies may introduce bias. Furthermore, incomplete data in certain studies precluded their inclusion in the analysis. The lack of these original data or relevant information limited our capacity to perform more comprehensive subgroup analyses. Although subgroup analyses were conducted, no significant sources of heterogeneity were identified, possibly due to insufficient exploration of potential heterogeneity. Thirdly, the relatively limited number of included studies, particularly those related to dNLR, restricted the scope of subgroup analyses. This limitation consequently impacted the precision of the pooled effect estimates. Moreover, variations in cut-off values across studies presented challenges for direct result comparisons. Such differences may have contributed to heterogeneity in patient stratification, introduced classification bias, and increased statistical noise. These factors may have undermined the stability of the correlations between LIPI, dNLR, LDH levels, and survival outcomes. This further impeded the translation of statistical associations into clinically actionable tools. For example, a higher cut-off value may result in fewer patients being categorized as having elevated LIPI, dNLR, or LDH levels, potentially modifying their prognostic significance. Future studies should prioritize establishing a standardized and unified cut-off value to optimize the prognostic utility of LIPI, dNLR, and LDH, while enhancing their consistency and reliability in clinical practice. Finally, despite most of the included studies adopting a treatment regimen involving immune checkpoint inhibitors combined with chemotherapy, variations in these regimens still existed, potentially contributing to increased heterogeneity in the pooled results. Future studies should further investigate the generalizability and robustness of LIPI, dNLR, and LDH across diverse immunotherapy regimens, including ICIs combined with chemotherapy or anti-angiogenic drugs.

## 5 Conclusion

This meta-analysis demonstrates that the baseline LIPI is a potent and novel prognostic factor for ES-SCLC patients receiving ICIs. Higher LIPI scores are significantly associated with a worse OS and PFS. This positions LIPI as a clinically valuable tool for prognostication in the context of ES-SCLC immunotherapy. While the individual components, LDH and dNLR, show associations with OS, LIPI integrates their information to provide a superior prognostic assessment. Therefore, validation in future high-quality prospective clinical studies is recommended to solidify its role in guiding ES-SCLC patient management.

## Data Availability

The raw data supporting the conclusions of this article will be made available by the authors, without undue reservation.
