# Supplementary material for: Prognostic value of baseline LIPI, LDH and dNLR in ES-SCLC patients receiving immune checkpoint inhibitors: a systematic review and meta-analysis
Source: Front Immunol. 2025 Sep 30;16:1640066. doi: 10.3389/fimmu.2025.1640066 (PMC12518118; doi:10.3389/fimmu.2025.1640066)
Supplement: Supplementary file 1 [file DataSheet1.docx]

Supplementary Figures


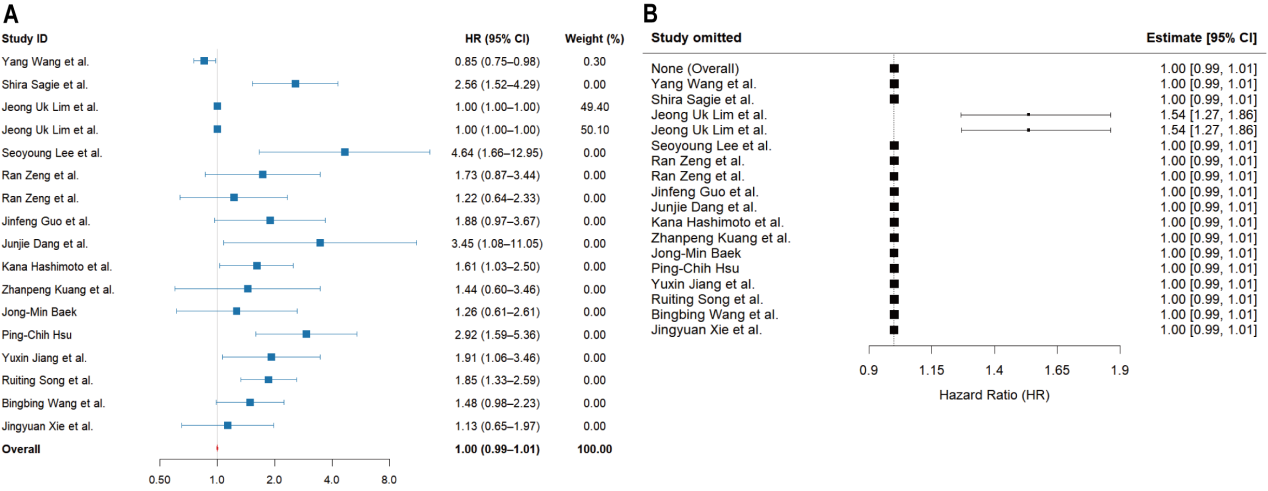


**Figure S1.** Forest plot and sensitivity analysis of baseline LDH Levels on overall HR and 95% CI of overall survival in patients with extensive stage small cell lung cancer treated with immune checkpoint inhibitors before excluding abnormal studies.


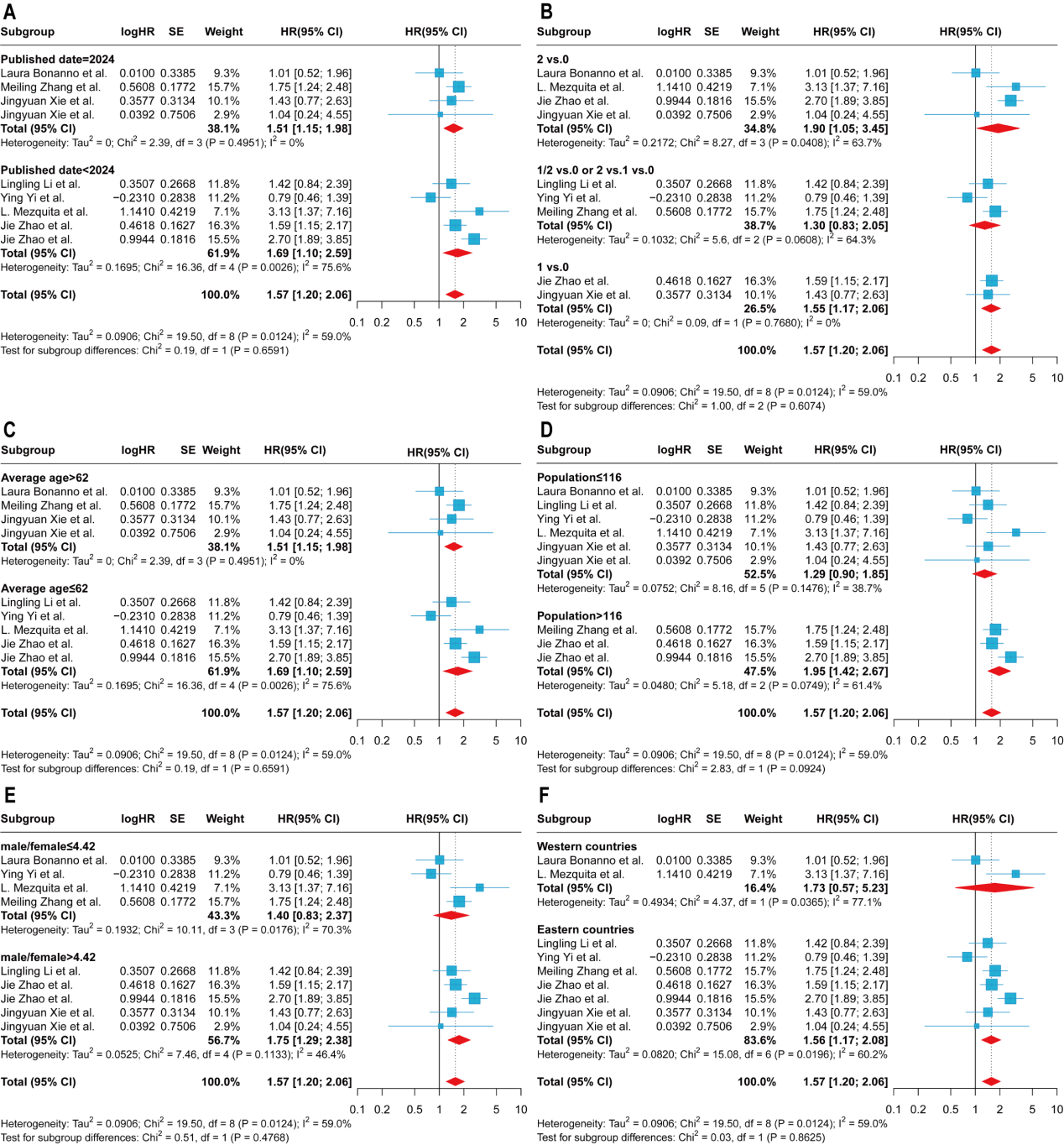


**Figure S2.** Subgroup analysis of the association between lung immune prognostic index and progression-free survival in patients with extensive-stage small cell lung cancer treated with immune checkpoint inhibitors based on published date(A), comparison methods(B),average age(C),population(D), sex ratio(E),country(F).


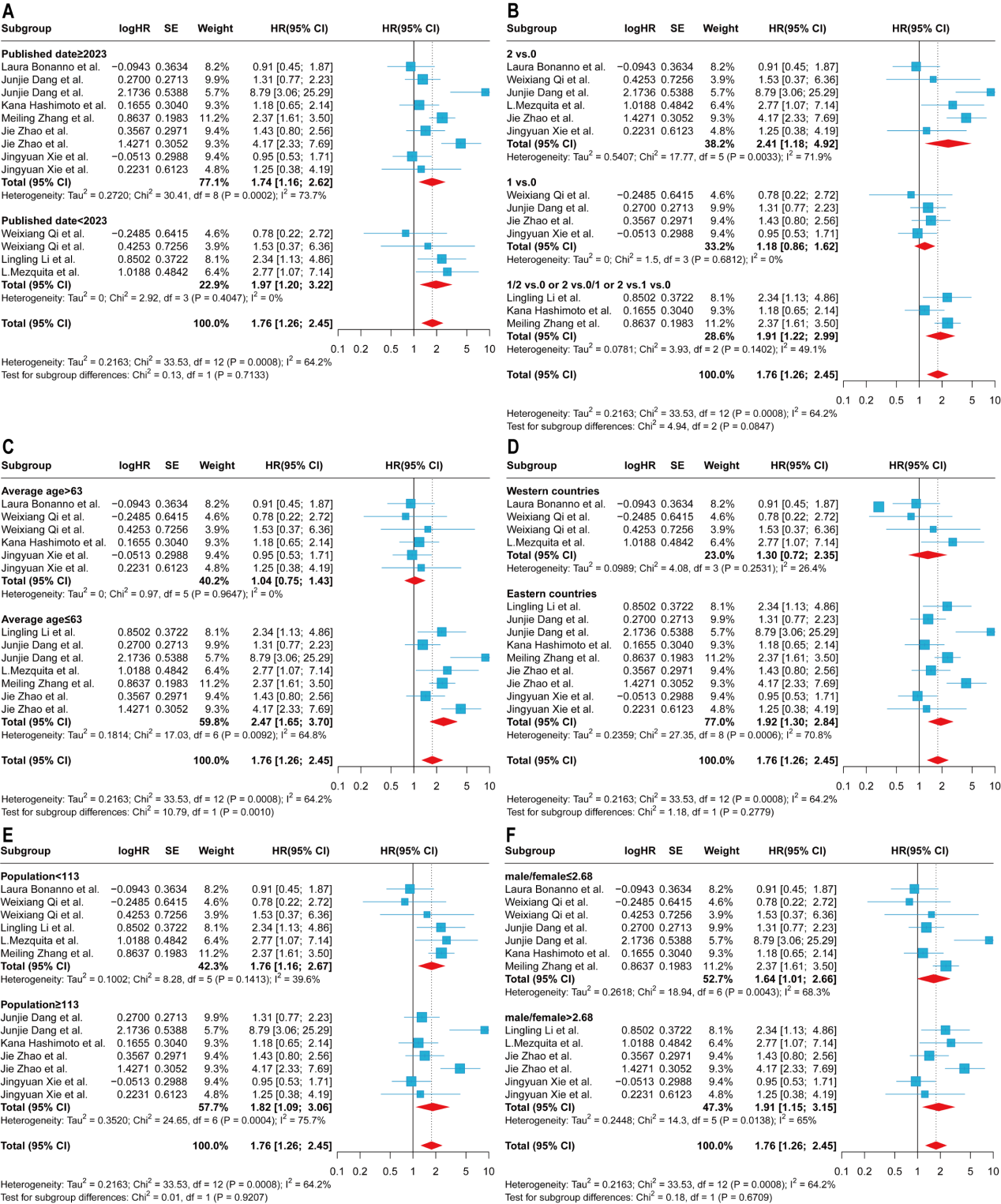


**Figure S3.** Subgroup analysis of the association between lung immune prognostic index and overall survival in patients with extensive-stage small cell lung cancer treated with immune checkpoint inhibitors based on published date(A), comparison methods(B),average age(C), country(D), population(E),sex ratio(F).


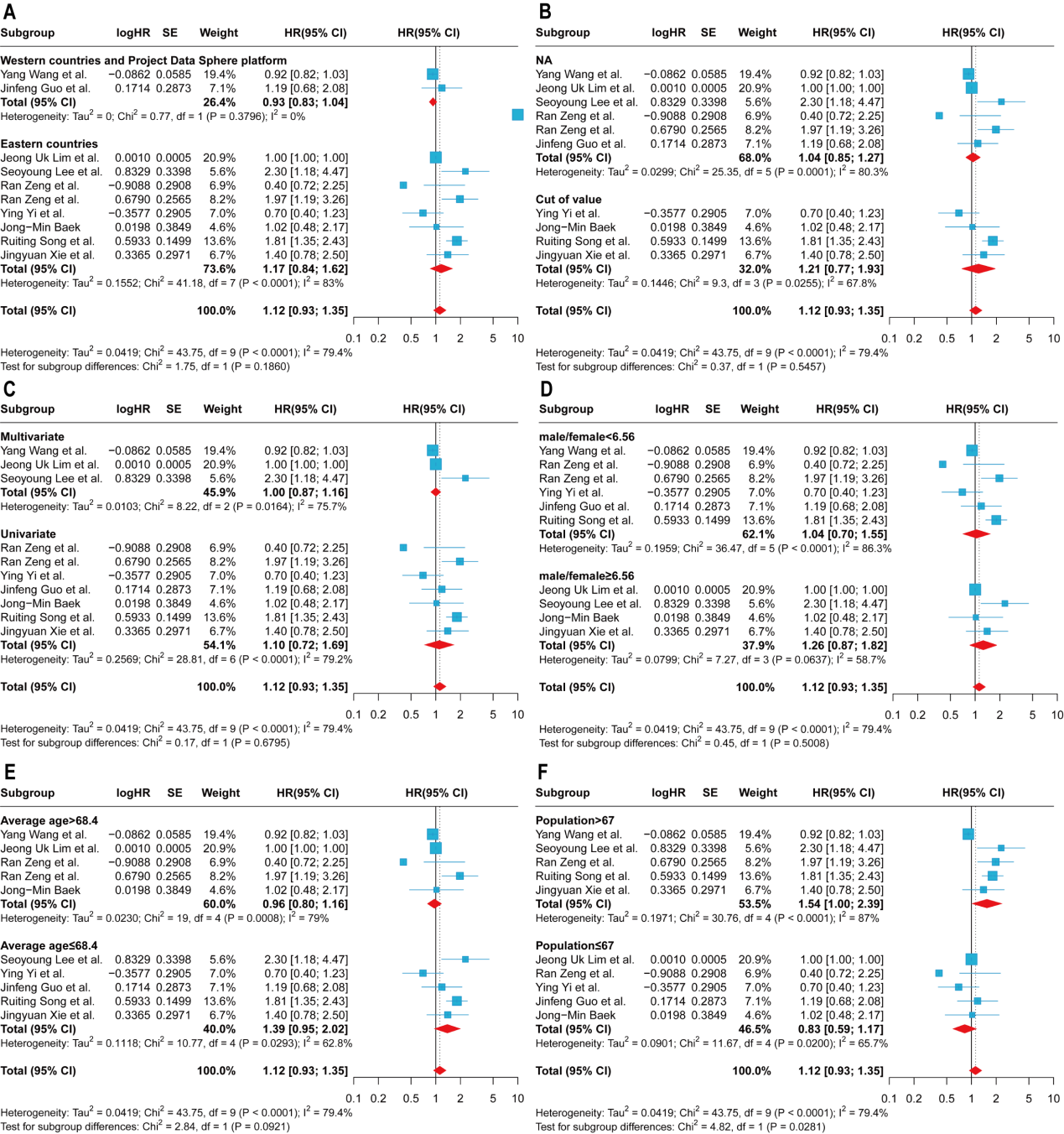


**Figure S4.** Subgroup analysis of the association between lactate dehydrogenase and progression-free survival in patients with extensive-stage small cell lung cancer treated with immune checkpoint inhibitors based on country(A),cut of value(B), univariate or multivariate(C), sex ratio(D), average age(E), population(F).


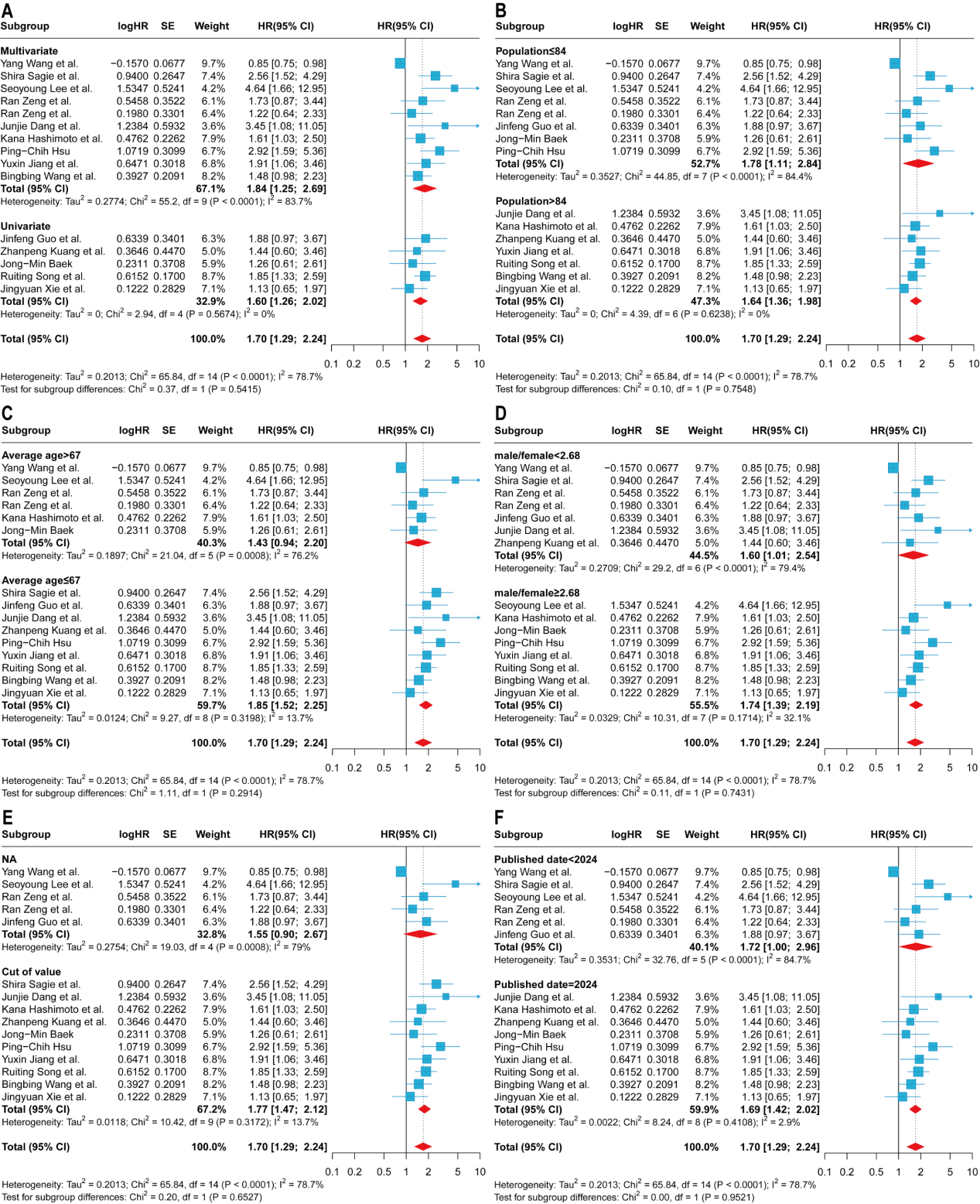


**Figure S5.** Subgroup analysis of the association between lactate dehydrogenase and overall survival in patients with extensive-stage small cell lung cancer treated with immune checkpoint inhibitors based on univariate or multivariate (A), population(B), average age(C), sex ratio(D), cut of value(E), published date(F).
