# Supplementary material for: Prognostic value of baseline LIPI, LDH and dNLR in ES-SCLC patients receiving immune checkpoint inhibitors: a systematic review and meta-analysis
Source: Front Immunol. 2025 Sep 30;16:1640066. doi: 10.3389/fimmu.2025.1640066 (PMC12518118; doi:10.3389/fimmu.2025.1640066)
Supplement: Supplementary file 2 [file Table1.docx]

**Table S1.** Details of searching strategy.

| **Database** | **Search strategy** |
| --- | --- |
| **PubMed** | (DNLR OR “derived neutrophil-to-lymphocyte ratio” OR “derived neutrophil lymphocyte ratio” OR LDH OR “lactate dehydrogenase” OR LIPI OR “lung immune prognostic index”) AND (“small cell lung cancer” OR “Small Cell Lung Carcinoma” OR “Small Cell Cancer Of The Lung” OR SCLC) NOT (“non-small cell lung cancer” OR NSCLC) AND (PD-1 OR PDL-1 OR CTLA-4 OR ICIs OR “immune checkpoint inhibitor” OR immunotherapy OR "Nivolumab" OR "Opdivo" OR "Pembrolizumab" OR "Keytruda" OR "Atezolizumab" OR "Tecentriq" OR "Durvalumab" OR "Imfinzi" OR "Avelumab" OR "adebrelimab" OR "Serplulimab" OR "Cemiplimab" OR "Envafolimab" OR "pidilizumab") |
| **COCHRANE and EMBASE** | (DNLR OR ‘derived neutrophil-to-lymphocyte ratio’ OR ‘derived neutrophil lymphocyte ratio’ OR LDH OR ‘lactate dehydrogenase’ OR LIPI OR ‘lung immune prognostic index’) AND (‘small cell lung cancer’ OR ‘Small Cell Lung Carcinoma’ OR ‘Small Cell Cancer Of The Lung’ OR SCLC) NOT (‘non-small cell lung cancer’ OR NSCLC) AND (PD-1 OR PDL-1 OR CTLA-4 OR ICIs OR ‘immune checkpoint inhibitor’ OR immunotherapy OR ‘Nivolumab’ OR ‘Opdivo’ OR ‘Pembrolizumab’ OR ‘Keytruda’ OR ‘Atezolizumab’ OR ‘Tecentriq’ OR ‘Durvalumab’ OR ‘Imfinzi’ OR ‘Avelumab’ OR ‘adebrelimab’ OR ‘Serplulimab’ OR ‘Cemiplimab’ OR ‘Envafolimab’ OR ‘pidilizumab’) |
| **Web of science** | TS=(DNLR OR derived neutrophil-to-lymphocyte ratio OR derived neutrophil lymphocyte ratio OR LDH OR lactate dehydrogenase OR LIPI OR lung immune prognostic index) AND TS=(small cell lung cancer OR Small Cell Lung Carcinoma OR Small Cell Cancer Of The Lung OR SCLC) NOT TS=(non-small cell lung cancer OR NSCLC) AND TS=(PD-1 OR PDL-1 OR CTLA-4 OR ICIs OR immune checkpoint inhibitor OR immunotherapy OR Nivolumab OR Opdivo OR Pembrolizumab OR Keytruda OR Atezolizumab OR Tecentriq OR Durvalumab OR Imfinzi OR Avelumab OR adebrelimab OR Serplulimab OR Cemiplimab OR Envafolimab OR pidilizumab) |

**Table S2** Raw data from forest plot (Figure 2A) with clinical outcomes of PFS

| Author | HR | 95%CI-low | 95%CI-high |
| --- | --- | --- | --- |
| Laura Bonanno et al. | 1.010 | 0.520 | 1.960 |
| Lingling Li et al. | 1.420 | 0.840 | 2.390 |
| Ying Yi et al. | 0.794 | 0.457 | 1.389 |
| L. Mezquita et al. | 3.130 | 1.370 | 7.160 |
| Meiling Zhang et al. | 1.752 | 1.238 | 2.480 |
| Jie Zhao et al. | 1.587 | 1.149 | 2.174 |
| Jie Zhao et al. | 2.703 | 1.887 | 3.846 |
| Jingyuan Xie et al. | 1.430 | 0.770 | 2.630 |
| Jingyuan Xie et al. | 1.040 | 0.240 | 4.550 |

**Table S3** Raw data from forest plot (Figure 2B) with clinical outcomes of OS

| Author | HR | 95%CI-low | 95%CI-high |
| --- | --- | --- | --- |
| Laura Bonanno et al. | 0.910 | 0.450 | 1.870 |
| Weixiang Qi et al. | 0.780 | 0.220 | 2.720 |
| Weixiang Qi et al. | 1.530 | 0.370 | 6.360 |
| Lingling Li et al. | 2.340 | 1.130 | 4.860 |
| Junjie Dang et al. | 1.310 | 0.770 | 2.230 |
| Junjie Dang et al. | 8.790 | 3.060 | 25.290 |
| Kana Hashimoto et al. | 1.180 | 0.650 | 2.140 |
| L.Mezquita et al. | 2.770 | 1.070 | 7.140 |
| Meiling Zhang et al. | 2.372 | 1.608 | 3.498 |
| Jie Zhao et al. | 1.429 | 0.800 | 2.564 |
| Jie Zhao et al. | 4.167 | 2.326 | 7.692 |
| Jingyuan Xie et al. | 0.950 | 0.530 | 1.710 |
| Jingyuan Xie et al. | 1.250 | 0.380 | 4.190 |

**Table S4** Raw data from forest plot (Figure 2C) with clinical outcomes of PFS

| Author | HR | 95%CI-low | 95%CI-high |
| --- | --- | --- | --- |
| Yang Wang et al. | 0.917 | 0.820 | 1.031 |
| Jeong Uk Lim et al. | 1.001 | 1.000 | 1.002 |
| Seoyoung Lee et al. | 2.300 | 1.180 | 4.470 |
| Ran Zeng et al. | 0.403 | 0.721 | 2.254 |
| Ran Zeng et al. | 1.972 | 1.193 | 3.261 |
| Ying Yi et al. | 0.699 | 0.395 | 1.235 |
| Jinfeng Guo et al. | 1.187 | 0.676 | 2.085 |
| Jong-Min Baek | 1.020 | 0.480 | 2.170 |
| Ruiting Song et al. | 1.810 | 1.350 | 2.430 |
| Jingyuan Xie et al. | 1.400 | 0.780 | 2.500 |

**Table S5** Raw data from forest plot (Figure 2D) with clinical outcomes of OS

| Author | HR | 95%CI-low | 95%CI-high |
| --- | --- | --- | --- |
| Yang Wang et al. | 0.855 | 0.752 | 0.980 |
| Shira Sagie et al. | 2.560 | 1.520 | 4.290 |
| Seoyoung Lee et al. | 4.640 | 1.660 | 12.950 |
| Ran Zeng et al. | 1.726 | 0.866 | 3.444 |
| Ran Zeng et al. | 1.219 | 0.638 | 2.327 |
| Jinfeng Guo et al. | 1.885 | 0.968 | 3.671 |
| Junjie Dang et al. | 3.450 | 1.080 | 11.050 |
| Kana Hashimoto et al. | 1.610 | 1.030 | 2.500 |
| Zhanpeng Kuang et al. | 1.440 | 0.600 | 3.460 |
| Jong-Min Baek | 1.260 | 0.610 | 2.610 |
| Ping-Chih Hsu | 2.921 | 1.591 | 5.362 |
| Yuxin Jiang et al. | 1.910 | 1.060 | 3.460 |
| Ruiting Song et al. | 1.850 | 1.330 | 2.590 |
| Bingbing Wang et al. | 1.481 | 0.983 | 2.231 |
| Jingyuan Xie et al. | 1.130 | 0.650 | 1.970 |

**Table S6**.Begg’test of the LIPI overall effect and 95%CI of different outcome

| Outcome | Begg’test | |
| --- | --- | --- |
|  | Z | P |
| PFS | -0.83 | 0.4042 |
| OS | 0.49 | 0.6255 |

**Table S7.**Begg’test of the LDH overall effect and 95%CI of different outcome

| Outcome | Begg’test | |
| --- | --- | --- |
|  | Z | P |
| PFS | -0.09 | 0.9287 |
| OS | 0.25 | 0.8046 |

**Table S8**.Begg’test of the dNLR overall effect and 95%CI of different outcome

| Outcome | Begg’test | |
| --- | --- | --- |
|  | Z | P |
| PFS | 1.36 | 0.1742 |
| OS | 1.36 | 0.1742 |
