# Supplementary material for: Prognostic value of baseline LIPI, LDH and dNLR in ES-SCLC patients receiving immune checkpoint inhibitors: a systematic review and meta-analysis
Source: Front Immunol. 2025 Sep 30;16:1640066. doi: 10.3389/fimmu.2025.1640066 (PMC12518118; doi:10.3389/fimmu.2025.1640066)
Supplement: Supplementary file 4 [file Table3.docx]

**Table S10.** Quality assessment of included studies through the Newcastle-Ottawa Quality Assessment Scale (NOS).

| **Items**  **Study** | **Selection** | | | | **Comparability** | **Assessment** | | | **Total score** |
| --- | --- | --- | --- | --- | --- | --- | --- | --- | --- |
|  | Representativeness of the exposed cohort | Selection of non-exposed cohort | Ascertainment of exposure | Demonstration that outcome of interest was not present at start of study | Comparability of cohorts on the basis of the design or analysis | Assessment of outcome | Was follow-up long enough for outcomes to occur | Adequacy of follow-up of cohorts |  |
| Laura Bonanno et al. | 1 | 1 | 1 | 1 | 2 | 1 | 1 | 0 | 8 |
| Wei‑Xiang Qi et al. | 1 | 1 | 1 | 1 | 2 | 1 | 1 | 1 | 9 |
| Yang Wang et al. | 1 | 1 | 1 | 1 | 2 | 1 | 1 | 0 | 8 |
| Shira Sagie et al. | 1 | 1 | 1 | 1 | 2 | 1 | 1 | 0 | 8 |
| Jeong Uk Lim et al. | 1 | 1 | 1 | 1 | 2 | 1 | 1 | 1 | 9 |
| Seoyoung Lee et al. | 1 | 1 | 1 | 1 | 2 | 1 | 1 | 1 | 9 |
| Ran Zeng et al. | 1 | 0 | 1 | 1 | 1 | 1 | 1 | 0 | 6 |
| Lingling Li et al. | 1 | 0 | 1 | 1 | 1 | 1 | 1 | 0 | 6 |
| Ying Yi et al. | 1 | 0 | 1 | 1 | 1 | 1 | 1 | 0 | 6 |
| Jinfeng Guo et al. | 1 | 1 | 1 | 1 | 1 | 1 | 1 | 0 | 7 |
| Junjie Dang et al. | 1 | 0 | 1 | 1 | 1 | 1 | 1 | 1 | 7 |
| Kana Hashimoto et al. | 1 | 0 | 1 | 1 | 1 | 1 | 1 | 1 | 7 |
| Zhanpeng Kuang et al. | 1 | 1 | 1 | 1 | 2 | 1 | 1 | 1 | 9 |
| L. Mezquita et al. | 1 | 0 | 1 | 1 | 1 | 1 | 1 | 0 | 6 |
| Meiling Zhang et al. | 1 | 1 | 1 | 1 | 2 | 1 | 1 | 1 | 9 |
| Jie Zhao et al. | 1 | 1 | 1 | 1 | 1 | 1 | 1 | 1 | 8 |
| Jong-Min Baek et al. | 1 | 0 | 1 | 1 | 1 | 1 | 1 | 0 | 6 |
| Julia Grambow-Velilla et al. | 1 | 0 | 1 | 1 | 1 | 1 | 1 | 0 | 6 |
| Ping-Chih Hsu et al. | 1 | 0 | 1 | 1 | 1 | 1 | 1 | 1 | 7 |
| Yuxin Jiang et al. | 1 | 1 | 1 | 1 | 1 | 1 | 1 | 1 | 8 |
| Ruiting Song et al. | 1 | 0 | 1 | 1 | 1 | 1 | 1 | 1 | 7 |
| Bingbing Wang et al. | 1 | 1 | 1 | 1 | 1 | 1 | 1 | 0 | 7 |
| Jingyuan Xie et al. | 1 | 1 | 1 | 1 | 1 | 1 | 1 | 1 | 8 |
